# Renal Arteriovenous Fistula

**DOI:** 10.5334/jbsr.2032

**Published:** 2020-01-31

**Authors:** Mats Van den broecke, Elke Vereecke, Pieter De Visschere

**Affiliations:** 1 University Hospital Ghent, BE

**Keywords:** Iatrogenic renal arteriovenous fistula, renal biopsy, color Doppler ultrasound

## Abstract

**Teaching Point:** Color Doppler ultrasound (US) is useful for screening for renal arteriovenous (AV) shunts, especially following renal biopsy, because of its convenience and minimally invasive nature.

## Case Presentation

**Figure 1:**
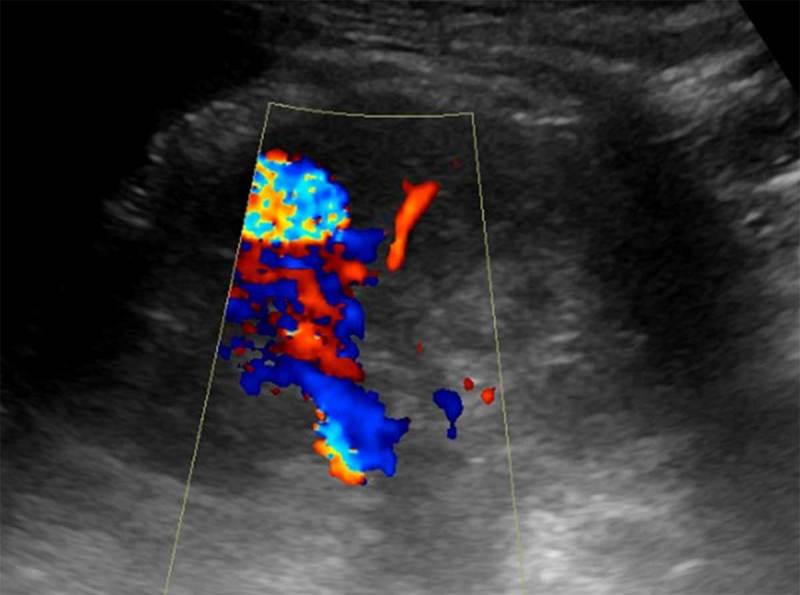
Color Doppler ultrasound of the left kidney: dilated tortuous blood vessels in the upper pole of the left kidney with pulsatile arterialized flow in the segmental draining vein. In the adjacent renal cortex, a connected rounded structure is noted with highly turbulent flow. Color Doppler ultrasound findings suggest an anomalous renal arteriovenous communication.

**Figure 2:**
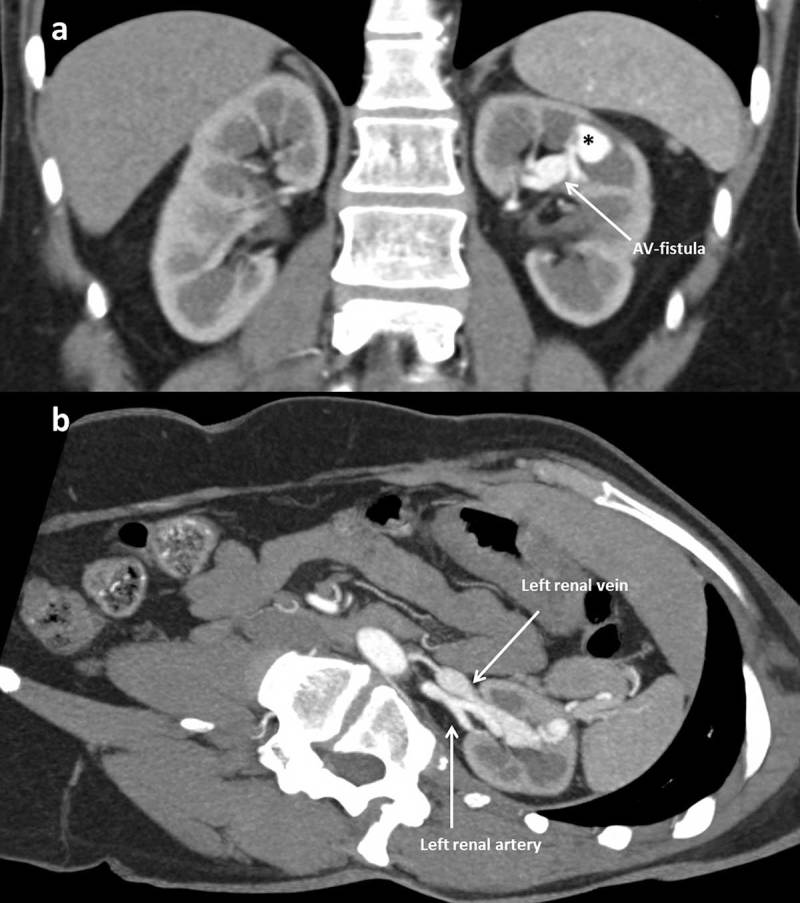
Abdominal CT scan, arterial phase, coronal slice **(a)** and reformatted axial slice **(b):** renal arteriovenous fistula with associated pseudoaneurysm (*) and early contrast opacification of the left renal vein in the arterial phase.

**Figure 3:**
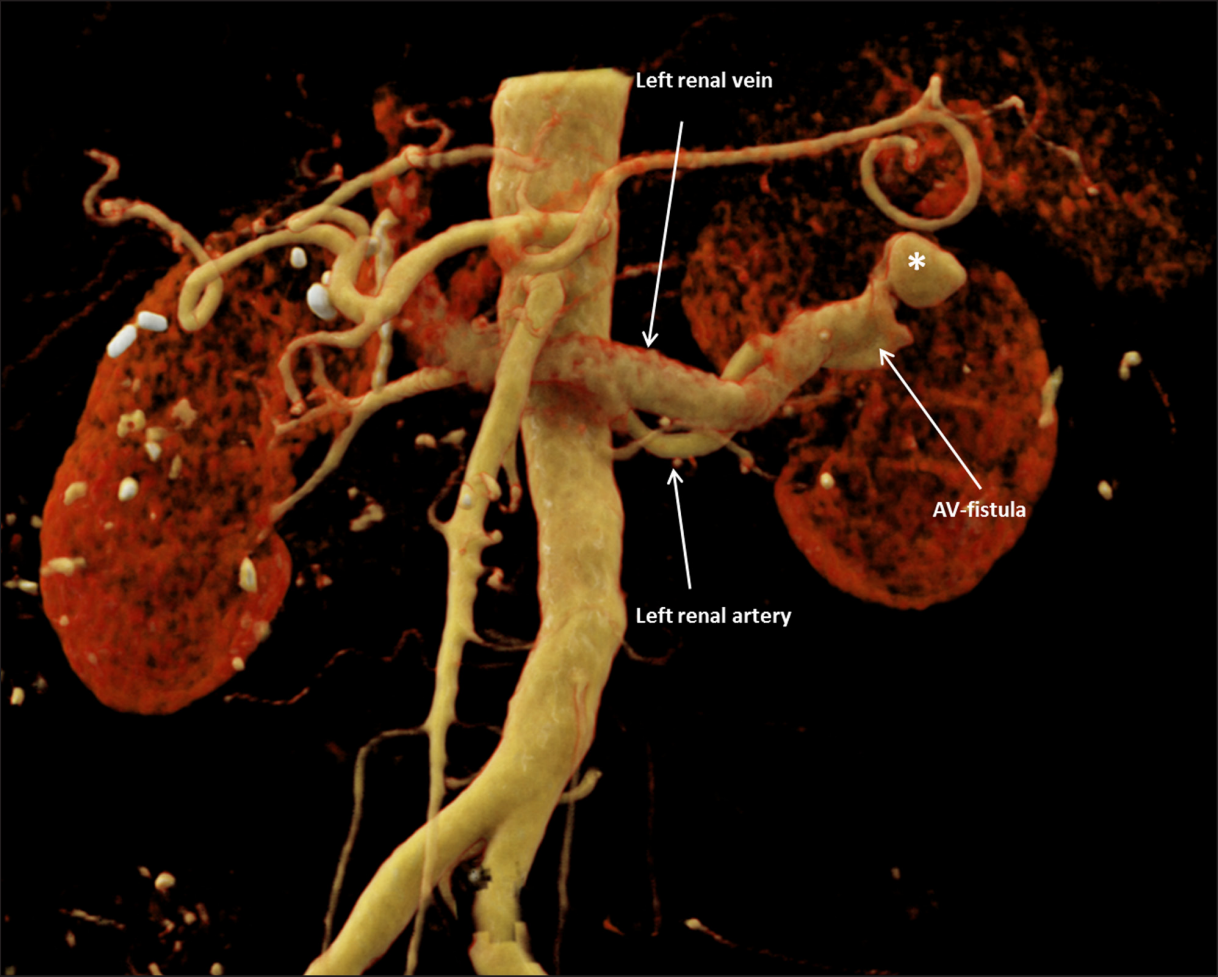
Volume rendering technique (VRT) of the arteriovenous fistula.

A patient with a history of liver transplantation was referred for ultrasound due to an acute on chronic renal insufficiency. Color Doppler ultrasound demonstrated dilated tortuous blood vessels in the upper pole of the left kidney with pulsatile arterialized flow in the segmental draining vein, suggesting an anomalous renal arteriovenous communication. In the adjacent renal cortex, a rounded structure with highly turbulent flow was noted in connection with this arteriovenous fistula (Figure 1). Computed tomography (CT) angiography confirmed a renal arteriovenous fistula with an associated pseudoaneurysm (asterisk on Figure 2a) and early opacification of the left renal vein (Figures 2b and 3). The arteriovenous fistula was probably iatrogenic as it was not seen on the ultrasound at the time of renal biopsy a few years earlier.

## Comment

Renal arteriovenous (AV) shunts, a rare pathologic condition, are divided into two categories, traumatic and nontraumatic, and can cause massive hematuria, retroperitoneal hemorrhage, pain and high-output heart failure. Traumatic renal AV shunts are caused by penetrating or blunt trauma, percutaneous or open biopsy, or surgery. The most common cause of traumatic renal AV shunts is iatrogenic injury, especially percutaneous renal biopsy. Several studies reported incidences of 7.4%–11% after renal biopsy. A majority of traumatic renal AV shunts due to renal biopsy are asymptomatic and resolve spontaneously within two years, but some can be symptomatic and require interventional treatment. They are usually solitary, involving a single direct communication between the renal artery and adjacent vein, so-called traumatic AV fistulas. Pseudoaneurysms occasionally coexist with traumatic renal AV shunts. Color Doppler US is useful for screening for renal AV shunts, especially following renal biopsy, because of its convenience and minimally invasive nature. A renal AV shunt appears as a mosaic pattern with a speckling of perivascular soft tissue caused by tissue vibration, reflecting a rapid flow rate. Increased flow velocity, decreased arterial resistance, and arterial waveforms in the renal vein can be observed with spectral analysis. Pre–contrast medium CT images can reveal renal hemorrhage, renal parenchymal calcifications and vascular wall calcifications. Aneurysms or varices related to a renal AV shunt are demonstrated as round or oval masses at pre-contrast CT. Post-contrast CT images typically show an early enhancement of the fistula and the ipsilateral renal vein. The preferred treatment is transcatheter embolization, which is a minimally invasive and effective treatment option. Potential complications include renal infarction, pulmonary embolism, and a potential risk of recanalization. Successful embolization of a renal AV shunt requires a complete occlusion of the shunted vessel while preventing the migration of embolic materials and preserving normal renal arterial branches. The selection of adequate techniques and embolic materials is based on the etiology and imaging angioarchitecture of the renal AV shunts [1].
